# Footwear and foot care knowledge as risk factors for foot problems in Indian diabetics

**DOI:** 10.4103/0973-3930.45269

**Published:** 2008

**Authors:** H. B. Chandalia, D. Singh, V. Kapoor, S. H. Chandalia, P. S. Lamba

**Affiliations:** Diabetes Endocrine Nutrition Management and Research Center (DENMARC), Mumbai, India

**Keywords:** Diabetic foot, education, footwear practices, risk factors

## Abstract

We assessed 300 diabetic and 100 age- and sex-matched controls for correlating foot wear practices and foot care knowledge and the presence of foot complications. A structured questionnaire evaluated the knowledge about foot care, type of footwear used, education level, association of tobacco abuse, and any associated symptoms of foot disease. Clinical evaluation was done by inspection of feet for presence of any external deformities, assessment of sensory function (vibration perception threshold, VPT), vascular status (foot pulses and ankle brachial ratio) and presence of any infection.

In the diabetes category, 44.7% patients had not received previous foot care education. 0.6% walked barefoot outdoors and 45% walked barefoot indoors. Fourteen (4.7%) patients gave history of foot ulceration in the past and comprised the high risk group; only 2 out of 14 had received foot care education, 6 gave history of tobacco abuse, 8 had symptoms of claudication, 9 had paresthesias, 2 walked barefoot indoors. Average duration of diabetes in the high-risk and low-risk diabetes group was 10.85 ± 6.53 and 9.83 ± 7.99 years, respectively. In the high- and low-risk diabetic groups, VPT was 19.57 ± 11.26 and 15.20 ± 10.21V (*P* < 0.02), ankle brachial ratio was 1.05 ± 0.19 and 1.14 ± 0.18 (*P* < 0.05), and the questionnaire scores was 40.8% and 57%, respectively.

In the diabetic and the control group, VPT was 15.62 ± 10.39 and 8.36 ± 3.61 V (*P* < 0.01), ankle brachial ratio was 1.14 ± 0.18 and 1.15 ± 0.12, and the questionnaire scores were 57% and 40.3%, respectively.

In conclusion, poor knowledge of foot care and poor footwear practices were important risk factors for foot problems in diabetes.

## Introduction

**Figure 1:**
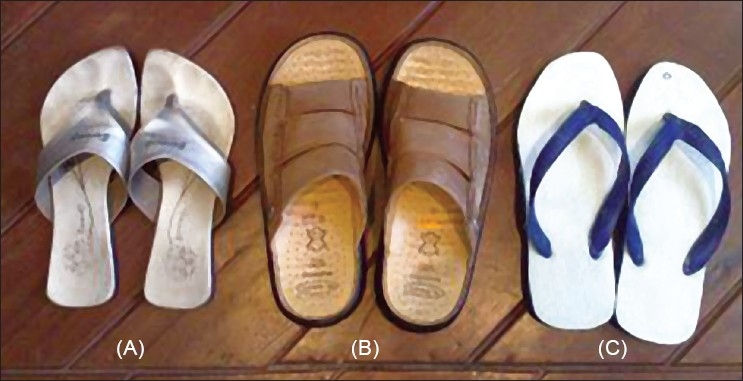
Commonly used footwear in India; all are without a heel counter. (A) Chappals with a grip-strap in the first web-space (B) Hawaiian slipper (C) Sandal

Diabetic foot syndrome is one of the common and most devastating preventable complications of diabetes mellitus (DM). The various factors contributing to this syndrome are peripheral sensory neuropathy, improper footwear, lack of patient knowledge about foot care and uncontrolled diabetes. In India, footwear practices vary widely.[1,2] Apart from a significant proportion of patients walking barefoot outdoors, a majority of Indians walk barefoot indoors. The custom of visiting religious shrines barefoot in a tropical country like India where the pavements or asphalt roads become very hot can lead to injury. Furthermore, use of inappropriate footwear like Hawaian chappals having a rubber sole, supported by a strap in the first inter-digital space, but no back strap predisposes to injury. A similar footwear, the Kolhapuri chappal, made of leather also exposes the feet to injury. Shoes when worn by either sex tend to be pointed and thus further expose the foot to injury [Figure 1]. Combining this with the practice of not wearing socks, particularly in Indian females can result in a hyperkeratotic and fissured heel or a callosity of the first interdigital space or injury to the great toe. Hence, this study was planned to study the impact of footwear practices and foot care and diabetes awareness on the development of diabetic foot disease.

## Materials and Methods

This prospective study was conducted in 300 Type 2 diabetics. All patients were > 18 years of age and gave written informed consent. They were evaluated for their knowledge about foot care and foot wear practices. Additionally, a detailed clinical and laboratory evaluation was done in this group to assess the presence of risk factors for foot problems and involvement of the feet. One hundred nondiabetic healthy relatives accompanying the diabetic patients coming to our centre were chosen as the control group.

A structured questionnaire was administered to both the groups. The questions elicited details of the type of foot wear preferred while walking outdoors and indoors, time of the day preferred for purchasing footwear, preference of the type of footwear for purchase, types of socks preferred and duration of use of new foot wear, care of feet at bedtime, practice followed while visiting a religious place, care of corn or callus, care of cut or blister or boil, cutting or trimming of toenails, care of dry skin, appreciation of symptoms like tingling, numbness and foot discoloration, pain on walking, and importance of white itchy patches on the feet.

In addition, a detailed history was taken regarding their general education level, duration of diabetes, foot care knowledge, and history of tobacco abuse, presence or absence of claudication, alteration in skin color and temperature and symptoms of neuropathy like numbness, tingling, burning, hyperesthesia and hypoesthesia. The patients were queried on any weakness of the ankle or foot, history of injury or infection of the feet, visit to a podiatrist, and a history of fungal infection.

Detailed foot examination was carried out. Any abnormality in the shape of the foot, redness or discoloration of skin, nail deformity or color change in the nails, prominent metatarsal head, clawing, hallux valgus, Charcot's deformity, callus, corns or foot ulcer were looked for. Fissuring of the foot, especially of the heel was sought for. Feet were then classified as per Wagner's classification.

The sensory function was assessed using the following quantitative testing procedures. (a) Vibration perception threshold (VPT) of both the feet was determined using a sensitometer (Dhansai Laboratory, Mumbai, India). (b) Touch pressure sensation was tested by Semmes Weinstein 10 gram monofilament, on the plantar and dorsal aspect of the foot and on the lateral malleolus of both the legs. (c) Ice packs and a test tube containing warm water were used to assess the thermal sensation.

The motor function was evaluated by testing for ankle reflex, power of the various muscle groups of the foot and by the presence of any wasting.

Sweating abnormalities and local temperature was assessed to determine autonomic function abnormality.

The vascular status was adjudged by the presence of pulsations of the lower limb (femoral, popliteal, dorsalis pedis and posterior tibial). The feet were examined for varicosities, presence of pallor or discoloration, edema and local temperature. Quantitative assessment was done by estimation of the ankle-brachial index by a Doppler study.

The footwear evaluation was done by categorizing the type of footwear into the following categories [Table 1]: (1) bare foot (2) open chappals or sandals with forking (3) straps without back support (4) straps with back support (5) leather shoes without laces, (6) leather shoes with laces, (7) sports or canvas shoes and (8) orthotic shoes. The footwear was also examined for well-fitting and ill-fitting status. The base of the footwear was looked for any unevenness. The front of the footwear was looked for its curvature, whether pointed or round or flat. The types of socks (cotton or synthetic, with or without seam) worn were inspected.

**Table 1:** Footwear practices (percent) observed in study cohort

Category	Diabetics outdoors % (*n* = 300)	Diabetics indoors % (*n* = 300)	Nondiabetics outdoors % (*n* = 100)	Nondiabetics indoors % (*n* = 100)
Barefoot	0.6	45	-	30
Open chappals/sandals with forking	13.3	48	12	51
Straps without back support	9.7	1	18	9
Straps with back support	23.3	5.7	41	10
Leather shoes without laces	27.0	-	19	-
Leather shoes with laces	9.7	-	-	-
Sports/canvas shoes	16.3	0.3	10	-
Orthotic shoes	-	-	-	-

Additionally, routine laboratory investigations including complete blood count, blood glucose fasting and 2-hour postprandial, HbA_1c_, serum creatinine, urine microalbumin, lipid profile and ECG were carried out. X-ray foot (anteroposterior and lateral view) was done in selected cases (Wagner 1 and above) for presence of bony deformities, osteomyelitis and Monckeberg's calcification.

## Results

We assessed 300 diabetic (M/F: 189/111, age: 52.2 ± 12.9 years) and 100 (M/F: 67/33, age: 47.1 ± 11.6 years) age- and sex-matched controls for correlating foot wear practices and diabetes awareness and the presence of foot complications. The average duration of diabetes was 9.96 ± 8.02 years (M/F: 9.73 ± 7.73/10.26 ± 8.52 years) in the study cohort.

Table 1 shows footwear practices followed by the study cohort. Unsafe footwear practices were prevalent in 46.9% of diabetics and 71% of nondiabetics outdoors. Significantly, 2 (0.6%) diabetics walked barefoot outdoors. None of the diabetics wore specially designed orthotic shoes.

**Figure 2:**
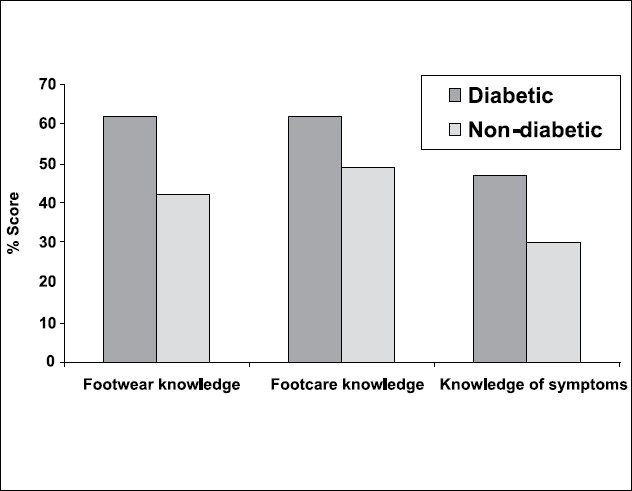
Questionnaire score in diabetics and non-diabetic controls

Assessment of the questionnaire revealed that diabetics had more awareness about footwear, foot care and knowledge of symptoms relating to diabetic foot than the nondiabetic controls [Figure 2]. In fact, controls could have received some education in foot care as relatives of diabetics. However, even in the diabetics the total average score was 57% indicating that there was scope for improving knowledge about prevention of diabetic foot disease.

55.3% of diabetics received specific foot care education compared to 4% of controls. 45.3% of diabetics were graduates, 49% were undergraduates and 5.7% had not studied beyond high school. The figures for controls were 46%, 29% and 25%, respectively. This indicates that the diabetics had received more formal education than the controls.

Predisposing conditions favoring diabetic foot were found in 81.3% of the diabetic group compared to 33% in the controls (*P* < 0.01). The most frequently encountered causes were calluses, corns and clawing deformity in 28.1% and tobacco abuse in 26.7% [Table 2].

**Table 2:** Predisposing conditions (percent) for diabetic foot in study cohort

	Diabetics			Controls		
		
	Total (*n* = 300)	Males (*n* = 189)	Females (*n* = 111)	Total (*n* = 100)	Males (*n* = 67)	Females (*n* = 33)
Fungus nails	3	3.2	2.7	2	-	6.1
Ingrowing nails	3.7	3.2	4.5	-	-	-
Clawing	9.7	9	10.8	-	-	-
Callus	14	13.2	15.3	6	6	6.1
Clawing + callus	2.7	3.2	1.8	-	-	-
Corn	1.7	2.6	-	8	11.9	-
Edema	10	5.8	17.1	1	-	3
Brittle nails	1	1	0.9	-	-	-
Hyperhidrosis	0.3	-	0.9	-	-	-
Anhidrosis	1.3	1.6	0.9	-	-	-
Hallux valgus	2.3	1.6	3.6	-	-	-
Prominent metatarsals	3.3	3.2	3.6	6	9	-
Weakness in the feet	1.7	1.6	1.8	-	-	-
Tobacco abuse	26.7	37	9	10	9	12.1
Total predisposing conditions	81.3	86.2	73	33	35.8	27.3

Clinical evidence of diabetic neuropathy was found variously when assessed by thermal sensation (1.3%), parasthesias (3%), motor dysfunction (1.3%) and Charcot's foot (0.3%). However, impaired vibration perception threshold (VPT) was observed in 31.3% of diabetics as compared to 8% in controls. The average VPT in the diabetic group was 15.66 ± 10.5 V compared to 8.91 ± 4.3 V in the controls (*P* < 0.01).

Evidence of ischemic foot (skin discoloration, absent pulsations, claudication, temperature change and varicosities) was found in 20.7% of diabetic and in 5% of controls. Abnormal ankle/brachial (A/B) ratio was found in 18 (6%) diabetics compared to 1(1%) in controls. The average A/B ratio in diabetics was 1.14 ± 0.18 as compared to 1.15 ± 0.12 in controls (*P* > 0.5).

The diabetic group was further subdivided into high-risk group (presence of past history of ulcers or presence of ulceration) and low-risk group [Table 3]. The high-risk group had a significantly high chance of developing foot problem as compared to the low-risk group, which in turn had a significantly high chance as compared to the controls.

**Table 3:** Comparison of high-risk and low-risk diabetics

	Low-risk diabetics (*n* = 286)	High-risk diabetics (*n* = 14)	Controls (*n* = 100)
Duration of DM (years)	9.83 ± 7.99	10.85 ± 6.53	-
Education in foot care (%)	57.3	14.3*	-
Tobacco abuse (%)	26.2	42.9*	10
Parasthesia (%)	43.4*	64.3*	7
Claudication (%)	-	57.1	-
VPT	13.6 ± 10.4**	19.6 ± 11.3*	8.36 ± 3.61
A/B ratio	1.14 ± 0.18	1.05 ± 0.19***	1.15 ± 0.12
Questionnaire score (%)	57	40.8	40.3

* *P* < 0.01;

** *P* < 0.02;

*** *P* < 0.05

## Discussion

Foot problems constitute a significant part of morbidity in diabetics in India.[1,2] There are some striking dissimilarities between foot problems in Western countries and India.[3] The etiology of the foot problems in India is primarily peripheral neuropathy, peripheral vascular disease being rare. It is really regrettable that surgical intervention or amputation is frequently required in our country for a neuropathic foot, which is entirely preventable. Hence, an insight into the factors leading to limb loss is essential.

In a neuropathic foot, deformity, skin problems (corn, callosity) and infection precipitate limb threatening complications. All these factors are eminently preventable or treatable. To seek timely advice and treatment depends upon patients general education and foot care education. The selection of appropriate footwear also is dependent on education.

This study reinforces the scope for improving foot care and footwear practices in the Indian diabetic and highlights the ignorance in foot care knowledge and practices, which contributes heavily to the susceptibility of the diabetic foot for injury and infection. It also signifies that if not checked in time, this may culminate into serious consequences like amputation of the affected foot.

The evaluation done by us brings forward the poor educational status of Indian diabetics, and this is supported by various other studies.[4–7] The contributing factors for this predisposition are busy clinical practice of diabetologists, who in turn spare little time for patient education regarding diabetes foot care, associated with reluctant and ignorant attitude of many patients to follow foot care practices for long. History of tobacco abuse in the form of smoking tobacco or chewing tobacco with betel leaf and gutkha (mixture of tobacco with betel nut and lime) is rampant in Indian diabetics, predisposing them to peripheral vascular conditions.[1,2,4,5] Poor motivation to maintain optimum glycemic control, negligent attitude towards injury, infection and other symptoms related to the feet leads to a delay in timely consultation. Though the average education in this study cohort was good, a noticeable apathy towards foot care and diabetes management was very apparent. Economic considerations may come in the way of obtaining regular follow up to prevent this complication.

This study emphasizes the high frequency of peripheral neuropathy and low frequency of peripheral vascular disease in the Indian diabetic. In this situation, efforts to institute good foot care practices are expected to be highly successful. An important component of these practices is selection of appropriate footwear. Our data bring out a very poor choice of footwear made both by our diabetics and controls [Table 1]. Vast majority of Indians use open footwear, called chappals. This has no heel counter and there is forking of toes by a divider. Frequency of foot deformities, corns, callosities and fungal infection is common even in controls [Table 2] and more so in diabetics. The situation is grim but the morbidity can be restricted or averted by modifying the above factors. There should be extra emphasis on patient education on diabetes and awareness of avoidable complications and their prevention by following good foot care practices.

All newly detected diabetics, as well as known diabetics should be educated about diabetes and its avoidable complications. A detailed foot examination should be done by the attending diabetologist at every visit of the diabetic patient to rule out neuropathy or vasculopathy. It is important on the part of the health care professional to identify the foot at risk. Diabetic patients with known history of tobacco abuse should be persuaded to quit tobacco. Although smoking is rather uncommon, particularly so in females, tobacco chewing is a rampant practice in both sexes. Patients with high-risk foot should be evaluated more frequently for development of additional risk of injury, infection and ulcer formation.

There is a need for a joint effort on part of the doctor and footwear industry and an active participation on behalf of the patient to receive education about foot care and improve their choice and selection of footwear so as to reduce foot problems.
